# Clinical Outcomes of Pars Plana Vitrectomy and Polytetrafluoroethylene (Gore-Tex) Scleral Fixation of a Monofocal Aspheric Intraocular Lens (Akreos AO60)

**DOI:** 10.1177/24741264241260094

**Published:** 2024-08-02

**Authors:** Amit V. Mishra, Rosanna Martens, Graeme K. Loh, Rizwan Somani, Mark D.J. Greve, Mark E. Seamone

**Affiliations:** 1 University of Alberta, Edmonton, AB, Canada; 2 Alberta Retina Consultants, Edmonton, AB, Canada

**Keywords:** secondary IOL, Akreos

## Abstract

**Purpose:** To evaluate the visual outcomes and complications with a polytetrafluoroethylene (Gore-Tex)-fixated intraocular lens (IOL) (Akreos AO60, Bausch + Lomb). **Methods:** Eyes with inadequate capsule support for in-the-bag IOL implantation had pars plana vitrectomy (PPV) and IOL placement at a single center. The main outcome measures were the postoperative visual acuity (VA) and complication rates. **Results:** During the study, 783 surgeries were performed. The mean visual acuity gain was 28.5 Early Treatment Diabetic Retinopathy Study letters (*P* < .01), with a mean time to best VA of 3.05 months. Statistical hypotony (intraocular pressure <6.5 mm Hg) was present in 214 cases (27.3%), and clinical features of hypotony were present in 46 cases. Five percent of the complications were directly related to the IOL. There were 3 cases of IOL opacification, 2 with silicone oil endotamponade and 1 with perfluoropropane endotamponade. **Conclusions:** PPV with Akreos IOL implantation was an effective technique for secondary IOL placement. Complications directly related to the IOL were uncommon.

## Introduction

Several techniques for managing aphakia in eyes without adequate capsule support have been described.^1–7^ In these cases, the surgical technique and intraocular lens (IOL) choice (eg, anterior chamber [AC] IOLs) scleral-fixated IOLs, and scleral-sutured IOLs, are based on the surgeon’s preference. With the decline in the placement of AC IOLs,^8^ vitreoretinal surgeons are using new techniques in eyes with inadequate capsule support. The overall rates of complications are low and do not vary significantly between techniques.^8,9^

The Akreos AO60 (Bausch + Lomb) is a 4-point scleral-fixated IOL that can be fixated using a polytetrafluoroethylene monofilament suture (CV-8 Gore-Tex).^10–12^ The IOL is monofocal with a 6.0 mm optic and is available in powers from 0 to +30.0 D. Theoretically, 4-point fixation prevents IOL tilt better than 2-point fixation. Polytetrafluoroethylene is preferred over polypropylene because of its resilience. Scleral-fixated IOLs and scleral-sutured IOLs also have benefits over AC IOLs in that they are located behind the iris and thus lower the risk for corneal decompensation over time.^8^

We present what to our knowledge is the largest series of secondary IOL surgery with the Akreos AO60 IOL and describe the visual outcomes and complications.

## Methods

A retrospective search of electronic patient records was performed at Alberta Retina Consultants. Data were collected between 2016 and 2023. Eyes having secondary IOL surgery with implantation of the Akreos AO60 IOL were included in the study. All cases had inadequate capsule support for in-the-bag IOL placement. All surgeries were performed by 1 of 8 retina specialists at the local academic hospital with trainee (fellow) assistance. The IOL power was calculated using standard biometry.

In all cases, pars plana vitrectomy (PPV) with a 25-gauge system (Alcon) was performed, with careful inspection of the retinal periphery for pathology. The IOL was inserted through a clear corneal incision and secured in place with a Gore-Tex suture. The inferior ends of the suture were passed superiorly subconjunctival to avoid the need for a conjunctival peritomy. The cornea was closed with 10-0 polyglactin (Vicryl) and the superior trocar sites with 8-0 polyglactin. Subconjunctival ceftazidime and dexamethasone were then administered. The Supplemental Video shows the surgical technique.

Baseline characteristics were obtained. All patients were included regardless of follow-up time. Further analysis of patients who had at least 3 months of postoperative follow-up was performed. The visual acuity (VA) before and after surgery and the time to best acuity were documented. Complications were identified and quantified. The intraocular pressure (IOP) 1 day postoperatively was recorded.

The primary outcome measure was the improvement in VA from baseline (before surgery). Other outcomes measures were the complication rates and rates of subsequent surgery.

Statistical analysis was performed using R software (version 3.6.2., R Foundation for Statistical Computing).

## Results

### Baseline Characteristics

The study comprised 783 eyes of 749 patients with a mean age of 73.8 years. Table 1 shows the baseline characteristics. The mean follow-up ranged from 1 to 72 months; 439 eyes had at least 3 months of follow-up (mean 15.8 months).

**Table 1.** Baseline Characteristics.

Characteristic	Value
Eyes (n)	783
Mean age (y)	73.6
Female sex	309
Mean follow-up (mo)	10.5
Surgical indication, n (%)	
Dislocated IOL	492 (63)
Dropped crystalline lens	120 (15)
Aphakia	115 (15)
UGH	40 (5)
PBK	11 (1)
Opacified IOL	5 (1)
Presenting lens status, n (%)	
PC IOL	490 (63)
Aphakia	142 (18)
Crystalline lens	97 (12)
SF IOL	42 (5)
AC IOL	12 (2)
Baseline VA (ETDRS), n (%)	22.3

Abbreviations: AC IOL, anterior chamber intraocular lens; ETDRS, Early Treatment Diabetic Retinopathy Study; IOL, intraocular lens; PBK, pseudophakic bullous keratopathy; PC IOL, posterior chamber intraocular lens; SF IOL, scleral fixated intraocular lens; UGH, uveitis–glaucoma–hyphema syndrome; VA, visual acuity.

Table 1 also shows the indications for surgery, the presenting lens status, and the best-corrected VA (BCVA) at baseline. The most common indication for surgery was a dislocated IOL followed by a retained lens fragment, aphakia, uveitis–glaucoma–hyphema (UGH) syndrome, pseudophakic bullous keratopathy, and IOL opacification. The most common presenting lens status was PC IOL followed by aphakia, crystalline lens, scleral-fixated IOL, and AC IOL. Of the patients, 209 had a previous vitrectomy, 37 had a previous globe rupture, and 36 had previous ocular trauma. The baseline BCVA of all patients was 22.3 Early Treatment Diabetic Retinopathy Study (ETDRS) letters and of patients who had at least 3 months of follow-up, 22.9 letters.

### Visual Acuity

**Figure 1.**
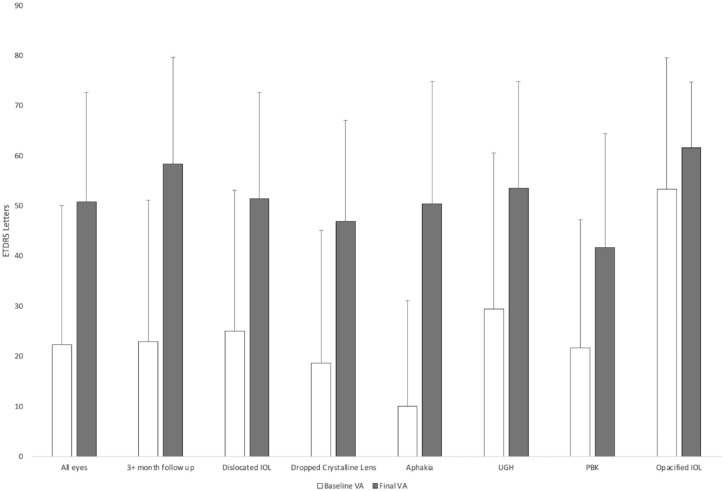
Mean baseline BCVA vs mean postoperative BCVA by indication for surgery. Abbreviations: BCVA, best-corrected visual acuity; ETDRS, Early Treatment Diabetic Retinopathy Study; IOL, intraocular lens; PBK, pseudophakic bullous keratopathy; UGH, uveitis–glaucoma–hyphema syndrome; VA, visual acuity.

Figure 1 shows the VA outcomes. In the entire cohort, the mean gain of ETDRS letters was 28.5 (*P* < .01). The mean time to the best VA (corrected) was 3.05 months postoperatively. There was a gain of 15 or more letters in 489 cases and a loss of 15 or more letters in 51 cases. The mean baseline VA was 12.5 letters and 53.9 letters, respectively; the difference was statistically significant (*P* < .01). For patients with at least 3 months of follow-up, the mean gain was 35.4 letters (*P* < .01) and the mean time to the best VA was 4.31 months.

Regarding VA by indication for surgery, there was significant mean VA gain in patients who required surgery for a dislocated IOL (26.4 letters), for aphakia (40.4 letters), for UGH (24.1 letters), and for a dislocated crystalline lens (28.3 letters) (all *P* < .01). There was no significant gain in patients requiring surgery for pseudophakic bullous keratopathy (20.0 letters; *P* = .09) or for an opacified IOL (8.33 letters; *P* = .44).

### Complications

Table 2 shows the complications. Overall, statistical hypotony (IOP <6.5 mm Hg) was present in 214 cases (27.3%) and clinical features of hypotony were present in 46 cases 1 day postoperatively. In patients with a minimum of 3 months of follow-up, the hypotony rate was 29% 1 day postoperatively, with 32 (7.3%) having clinical features of hypotony. The rate of postoperative hypotony using 5 mm Hg as a cutoff was 13.6%.

**Table 2.** Complications.

Complication	Number (%)
Statistical hypotony (IOP < 6.5 mm Hg)	214 (27.3)
Clinical features of hypotony	46 (5.9)
Elevated IOP	93 (11.9)
CME	90 (11.5)
Retinal detachment	15 (2.0)
Vitreous hemorrhage	15 (2.0)
Endophthalmitis	8 (1.0)
IOL specific	
Exposed suture	8 (1.0)
IOL dislocation	6 (0.8)
IOL opacification	3 (0.4)

Abbreviations: CME, cystoid macular edema; IOL, intraocular lens; IOP, intraocular pressure.

The next most common complication was elevated IOP (11.9%) followed by cystoid macular edema (CME) (11.5%) and vitreous hemorrhage (2.0%). Postoperative hypotony was present in 0.30% of eyes with vitreous hemorrhage. Eight eyes (1.0%) developed endophthalmitis. The rate of IOL-specific complications was 5%; they included an exposed polytetrafluoroethylene suture (1.02%), a dislocated IOL (0.77%), and IOL opacification (0.38%). The mean time to polytetrafluoroethylene suture exposure was 14.5 months.

### Additional Surgery

Twenty-seven eyes required further vitrectomy surgery. The indications included endophthalmitis (n = 6), retinal detachment (RD) (n = 15), choroidal detachment (n = 3), epiretinal membrane (n = 2), and hyphema (n = 1). Two eyes with endophthalmitis (0.33%) had hypotony in the postoperative period. There was 1 late case of endophthalmitis associated with suture exposure; all the other cases occurred in the immediate postoperative period (mean 1.3 months).

**Figure 2.**
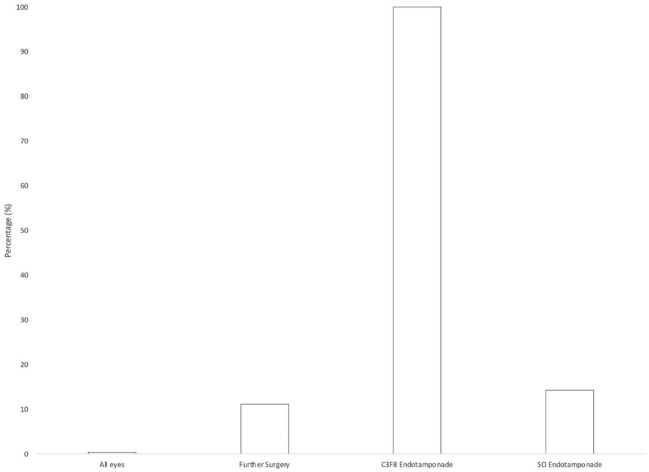
Intraocular lens opacification rate in all eyes (N = 783), in eyes that required further surgery (n = 27), in eyes that required C_3_F_8_ endotamponade (n = 1), and in eyes that required SO endotamponade (n = 14). Abbreviations: C_3_F_8_, perfluoropropane; SO, silicone oil.

In 15 of the secondary vitrectomy surgeries, fluid–air exchange was performed and an endotamponade was used; the endotamponade was silicone oil (SO) (5000 centistoke) in 14 cases and perfluoropropane (C_3_F_8_) in 1 case. The mean time to SO removal was 6.5 months. There were 3 cases of IOL opacification, 2 in eyes with an SO endotamponade and 1 in an eye with a C_3_F_8_ endotamponade (Figure 2). The time to opacification was 3 months and 1 month, respectively. In the patient with IOL opacification and C_3_F_8_ endotamponade, the VA remained stable at 65 letters; thus, the IOL was left in situ after discussion with the patient. The VA with IOL opacification was 5 letters and 0 letters in the 2 patients with SO endotamponade. Both IOLs were explanted and replaced with a new Akreos IOL. The VA improved to 55 letters and 30 letters, respectively.

## Conclusions

This study evaluated a large series of patients (783 surgeries) who had PPV and Akreos AO60 IOL implantation during secondary IOL surgery. We found significant improvements in VA, with a gain of 28.5 ETDRS letters in the entire cohort and of 35.4 letters in patients with more than 3 months of follow-up. These gains are within the range of those reported in the literature. A recent review of 97 cases with Akreos IOL placement showed a gain of approximately 20 letters.^13^ Smaller studies have shown greater gains of approximately 50 letters after surgery.^10,12,14^ Our study likely underestimated visual gains because a formal refraction is not performed in our clinic postoperatively. The most significant gains in our study were in the aphakia group, while the group with an opacified IOL had the smallest VA change.

Our study is unique in that we assessed the time to the best acuity measured after secondary IOL surgery. The mean time to the best measured VA in the clinic was 3.05 months postoperatively. In patients with a longer follow-up, the mean time to the best VA was 4.31 months. We would expect VA to improve approximately 2 to 3 months postoperatively given that the clear corneal incision was closed with a 10-0 polyglactin suture. The time to absorption of polyglactin is approximately 56 to 70 days, which would reduce astigmatism.^15^ These data can help clinicians give patients better estimates of the timeline to visual improvement after surgery.

In our study, statistical hypotony was present in 27.3% of cases; however, only 5.9% had clinical features of hypotony. This rate of hypotony is higher than what has been reported in the literature. In smaller case series, the rate of hypotony was between 3% and 13%.^11–14^ Most of these series defined hypotony as an IOP less than 6 mm Hg or less than 5 mm Hg; therefore, we may have captured a larger population given our higher cutoff. The value of 6.5 mm Hg was selected because it isolates IOP more than 3 standard deviations below the mean. In addition, we quantified hypotony as any IOP below 6.5 mm Hg starting on postoperative day 1. Using stricter cutoff rates, in line with the published literature, the incidence of hypotony in our study was 19.9% with an IOP cutoff of 6 mm Hg and 13.5% with an IOP cutoff of 5 mm Hg.

Postoperative hypotony in our study was likely related to the multiple incisions required. All eyes required a clear corneal incision for IOL insertion, four 25-gauge sclerotomies for the polytetrafluoroethylene monofilament suture, and another 25-gauge sclerotomy for infusion. In all cases, the superior sclerotomies were sutured with 8-0 polyglactin to reduce the rate of hypotony. The main corneal incision was closed with interrupted 10-0 polyglactin sutures. Given the hypotony rates, suturing the inferior sclerotomies that are used for the Gore-Tex could be performed to lower the rate. Incision size is also likely related to the postoperative rates of hypotony. Some studies use a 27-gauge system, which would likely lower postoperative hypotony rates. However, most eyes in our study did not have clinical features of hypotony or require intervention. Only 1 case with choroidal detachment secondary to surgery required further surgery. All other cases resolved within 1 month of surgery without intervention.

Other complications included elevated IOP, CME, vitreous hemorrhage, hyphema, and RD. The endophthalmitis rate was 1.02%. Endophthalmitis rates after 25-gauge vitrectomy range from 0.03% to 0.84% in the literature.^16^ Rates are likely higher after secondary IOL surgery because of the higher rates of hypotony postoperatively and the increased number of incisions required. In our study, the rates of IOL-specific complications, including suture erosion and IOL dislocation, were similar to those the literature.^13^

Further vitrectomy was required in 27 cases (3.4%) in our cohort. This is lower than the rate reported by Pardini et al (8.9%).^13^ Of these cases, 15 had fluid–air exchange intraoperatively and use of an endotamponade. Opacification has been reported with fluid–air exchange and endotamponade use.^17–20^ Recently, there have been reports of IOL opacification without further surgery.^21^ There were 3 cases (20%) of postoperative opacification in our cohort. This is slightly lower than rates reported in case series of patients who required secondary vitrectomy surgery.^19,20^ In our study, IOL opacification occurred with gas endotamponade and SO endotamponade, similar to findings in other studies.^17-20^ The time to opacification was 3 months with SO and 1 month with C_3_F_8_ gas. The case of opacification secondary to C_3_F_8_ was mild, and the IOL was left in situ because the patient maintained good VA. Both cases of opacification with SO had significant vision loss; thus, the IOL was explanted and replaced with a new Akreos AO60. The mechanism of opacification is still not well understood. Several theories have been proposed, including IOL dehydration during air–fluid exchange and persistent inflammation.^13^

The main strength of our study is the large sample. To our knowledge, this is the largest series of secondary IOL surgeries using the Akreos IOL. In addition, the follow-up period for many of our patients was significant. Limitations of our study include its retrospective design and loss to follow-up. A major limitation is the lack of a formal refraction after surgery. This was a real-world study in a busy retina practice; thus, we relied on pinhole VA to assess the visual outcomes after surgery. All patients are referred to their optometrists for formal refraction after the initial postoperative period. It has been shown that pinhole acuity is not equivalent to VA with correction after formal refraction or autorefraction.^22^ In our study, the visual gains may represent an underestimation of the true improvement one might find with a formal refraction. Another limitation is the difference in total follow-up times. Given the large catchment area of our practice, many patients are followed by their local ophthalmologist/optometrist after the immediate postoperative period. Standardized follow-up would better identify more long-term outcomes of the surgical technique.

Our study results lead to further research questions. A prospective study of the results of the technique with refraction would be beneficial for further outcomes assessment. Also, given the multiple current techniques used for the surgical correction of aphakia in eyes without capsule support, a prospective study comparing scleral-sutured IOL techniques and scleral-fixated IOL techniques would help determine whether there was an ideal method with regard to visual outcomes and complications. Our results have implications for clinical practice. Given our findings, more informed discussions can be had with patients regarding the timeframe for visual improvement and the overall prognosis.

In summary, we present the postoperative outcomes in a large series of secondary IOL surgery using the Akreos AO60 IOL with Gore-Tex scleral fixation. We found significant visual gains. Common complications included hypotony and elevated IOP in the early postoperative period. Complications related to the IOL were rare, with only 3 cases of IOL opacification.
